# Telehealth Psychological Interventions for Rural Caregivers: Improving Care to Persons With Dementia

**DOI:** 10.1093/geroni/igaa057.2283

**Published:** 2020-12-16

**Authors:** Joleen Sussman, Nikhil Banerjee, James Winslow

**Affiliations:** Veterans Affairs, Aurora, Colorado, United States

## Abstract

Geropsychologists are well-suited to assess dementia, assist caregivers in understand the disease and associated behavioral changes and ways to cope with their loved one’s disease. However, majority of these services are offered during in-person clinic appointments in urban settings. This study aims to describe the utilization and potential benefits of providing dementia education telehealth services to Veterans and their families residing in rural mountain and plain areas of Colorado. Psychological intervention was provided via telehealth from the primary VA hospital to small community clinics or to Veterans homes via video mobile application. The present study provides demographics of participants who elect this service and discusses how these challenge ageism and other biases relate to technology use. Further, we examined how engagement in this intervention may impact utilization of geriatric and extended care services as well as use of primary care, emergency room visits, and use of anti-psychotic medications.

